# Efficacy of Cryopreserved Amniotic Membrane Allograft in the Management of Refractory Chronic Venous Leg Ulcers: A Randomized Controlled Trial

**DOI:** 10.7759/cureus.104015

**Published:** 2026-02-21

**Authors:** Mohit Naren Kondapalli, Sanjay C Desai, Sandeep Mani Kumar Jakka, Suhas S Gowda

**Affiliations:** 1 Department of Vascular Surgery, Ramaiah Medical College, Bengaluru, IND

**Keywords:** amniotic membrane allograft, biological dressing, chronic venous leg ulcers, cryopreserved amnion membrane allograft, regenerative medicine, vascular surgery, wound healing

## Abstract

Background and aims

Chronic venous leg ulcers present a clinical challenge due to persistent inflammation, proteolytic imbalance, and senescence of the extracellular matrix. Dehydrated amniotic products are utilized; however, cryopreserved amniotic membrane retains the native heavy chain hyaluronic acid and viable growth factors that are often altered during heat dehydration processing. This study evaluated the efficacy of cryopreserved amniotic membrane allografts in promoting wound closure and reducing pain in patients with refractory venous ulcers compared to standard compression therapy.

Methods

A prospective, single-center, randomized controlled trial was conducted. Sixty-four patients with venous ulcers persisting for more than eight weeks were randomized (1:1). The intervention group (n=32) received topical application of culture-confirmed cryopreserved amniotic membrane alongside standard multi-layer compression therapy. The control group (n=32) received standard compression therapy alone. Wounds were assessed weekly by blinded independent evaluators. The primary efficacy endpoint was the complete wound closure rate at 45 days. Secondary outcomes included Kaplan-Meier survival analysis for time-to-healing, microbiologically confirmed infection rates, and pain reduction measured by the Visual Analog Scale (VAS). All analyses were conducted on an intention-to-treat (ITT) basis.

Results

The intervention group achieved a higher rate of complete wound closure (81.25%) compared to the control group (46.88%) at 45 days (Risk Difference: 34.4%, 95% CI: 12.4% to 56.3%; p=0.004). Kaplan-Meier survival analysis indicated a lower median time to complete epithelialization in the intervention group (36 days, 95% CI: 31-41) versus the control group (78 days, 95% CI: 71-85) (Log-rank test, p<0.001). Patients in the intervention group reported lower VAS pain scores by day 14. The incidence of confirmed wound infection was 6.25% in the intervention group versus 28.12% in the control group (Risk Difference: 21.9%, 95% CI: 4.4% to 39.4%; p=0.02).

Conclusion

Cryopreserved amniotic membrane promotes wound closure and reduces pain in refractory chronic venous leg ulcers. Its application serves as a biological adjunct to standard compression therapy in the management of hard-to-heal wounds.

## Introduction

Chronic venous leg ulcers affect approximately 1% of the adult population and are associated with morbidity, often leading to prolonged hospitalization and altered quality of life [1]. The pathophysiology of these non-healing wounds involves a prolonged inflammatory phase, elevated levels of matrix metalloproteinases (MMPs), and a deficiency of endogenous growth factors [2]. Standard compression therapy exhibits variable failure rates, prompting the investigation of biological therapies to address the stalled wound state [3].

Human amniotic membrane, comprising the amnion and chorion layers of the placenta, serves as a biological dressing [4]. It functions as a biological scaffold containing types I, III, IV, and V collagen, fibronectin, and laminin [5]. It is also a reservoir of bioactive molecules, including vascular endothelial growth factor, transforming growth factor-beta, and tissue inhibitors of metalloproteinases [6]. In theory, these components modulate inflammation, promote angiogenesis, and facilitate the recruitment of stem cells to the wound bed [7].

While the utility of dehydrated amniotic membrane is documented, evidence regarding the efficacy of cryopreserved amniotic membrane specifically for vascular ulcers is emerging [8,9]. Unlike dehydrated forms, tissue cryopreserved at -80°C retains the native histological structure and the integrity of its heavy-chain hyaluronic acid complex [10]. A barrier to utilizing cryopreserved tissue in the Indian demographic involves the logistical requirement for rigorous microbiological screening and cold-chain supply.

Evidence indicates that cryopreserved amniotic membrane provides distinct biological activity in complex clinical scenarios [11]. There is currently limited data comparing this modality against the standard of care in the Indian population. The primary objective of this study was to evaluate the efficacy of cryopreserved amniotic membrane allografts in promoting wound closure and reducing pain in patients with refractory chronic venous leg ulcers.

## Materials and methods

Study design and ethical considerations

This prospective, single-center, randomized controlled trial was conducted at the Department of Vascular and Endovascular Surgery, Ramaiah Medical College, Bengaluru, India. The trial was registered prospectively with the Clinical Trials Registry - India (CTRI Ref No: REF/2024/12/095695) prior to patient enrollment. The study protocol was approved by the Institutional Ethics Committee (Ethical Clearance No: MSRMC/EC/SP-10/08-2024 dated August 30, 2024) and adhered to the principles of the Declaration of Helsinki. Informed written consent was obtained from all participants.

Participants

The study included 64 patients aged 18 years and above presenting with chronic venous ulcers. Inclusion criteria were venous ulcers (classified as C6 according to the Clinical, Etiological, Anatomical, and Pathophysiological (CEAP) classification system) persisting for more than eight weeks and measuring greater than 10 cm², with an ankle-brachial index between 0.9 and 1.2. Exclusion criteria included acute wounds, active infection (e.g., osteomyelitis), uncorrected arterial insufficiency, uncontrolled diabetes (HbA1c >8%), malignancy, or pregnancy.

Sample size calculation

A formal a priori power analysis was not conducted. The sample size was determined based on the availability of eligible patients presenting to the tertiary care center during the defined study period (convenience sampling). However, a post-hoc power calculation (assuming an alpha level of 0.05 and the observed closure rates) indicated that a sample size of 64 patients (32 per arm) provided >80% power (beta = 0.20) to detect the 34% difference in the primary outcome of wound closure at 45 days.

Randomization and blinding

Participants were randomized into two groups (1:1 allocation ratio) using a computer-generated block randomization sequence. Allocation concealment was maintained using sequentially numbered, opaque, sealed envelopes (SNOSE), which were opened strictly at the time of intervention. Due to the visual appearance of the allograft, blinding of the operating surgeons and patients was not feasible. To mitigate observer bias, wound area measurements and clinical assessments were performed by an independent outcome assessor who was blinded to the treatment allocation. Furthermore, wound measurements were independently verified using digital planimetry software.

Tissue preparation and shelf life

Placentas were harvested from seronegative donors undergoing elective cesarean sections. Under aseptic conditions, the amnion was separated from the chorion and treated with an antibiotic-saline solution. Microbiological safety was confirmed via negative bacterial and fungal cultures prior to processing. The tissue was mounted on nitrocellulose paper, placed in a 1:1 mixture of sterile glycerol and Dulbecco’s Modified Eagle Medium (DMEM) acting as a cryoprotectant, and cryopreserved at -80°C. Prior to clinical application, the graft was thawed. Validation protocols confirmed that this cryopreservation method maintains structural integrity and cellular viability for up to six months.

Intervention

The intervention group (n=32) underwent sharp debridement followed by the topical application of the thawed cryopreserved amniotic membrane. The membrane was applied with the stromal side facing the wound bed to facilitate cellular adhesion. The graft was secured with a non-adherent secondary dressing and a four-layer compression bandage system. The protocol utilized a baseline application; however, the membrane was sequentially reapplied if the initial graft fully resorbed into the wound bed or if healing trajectories plateaued beyond 20 days. The control group (n=32) received identical sharp debridement followed by standard saline dressings and four-layer compression therapy.

Outcome measures

Wounds were clinically assessed weekly for a 12-week follow-up period to monitor healing, graft integration, and adverse events. The primary efficacy endpoint was the complete wound closure rate at 45 days. This intermediate milestone was selected based on wound healing kinetics; early area reduction within the four-to-six-week window serves as a validated prognostic indicator for chronic ulcer healing trajectories. Evaluating closure exclusively at the 12-week mark risks statistically masking early variance, as standard compression care achieves eventual closure over a protracted timeline.

Secondary outcomes

Secondary outcomes included time to complete epithelialization, pain assessment using the Visual Analog Scale (VAS) [12], and clinical infection rates. To ensure diagnostic accuracy, clinical infection was predefined as the presence of purulence, expanding erythema, or malodor, and required microbiological confirmation via positive swab cultures prior to the initiation of systemic antibiotic therapy.

Statistical analysis

Data analysis was performed using IBM SPSS Statistics for Windows, Version 26.0 (IBM Corp, Armonk, NY). All analyses were conducted on an Intention-to-Treat (ITT) basis. Missing data for the primary outcome were handled using the Last Observation Carried Forward (LOCF) method. Continuous variables were assessed for normality using the Shapiro-Wilk test. Normally distributed parametric data (e.g., age, baseline wound area) were presented as mean ± standard deviation (SD) and compared using the independent Student’s t-test.

Non-normally distributed continuous data (e.g., VAS scores) were presented as median (interquartile range) and compared using the Mann-Whitney U test. Categorical variables (e.g., complete closure rates, infection rates) were expressed as frequencies and compared using Fisher’s exact test. For time-to-event data, survival analysis was conducted using Kaplan-Meier curves, and differences were evaluated using the Log-rank test. Differences in proportions and medians were reported with 95% Confidence Intervals (CI). A two-sided p-value <0.05 was considered statistically significant.

## Results

**Figure 1:**
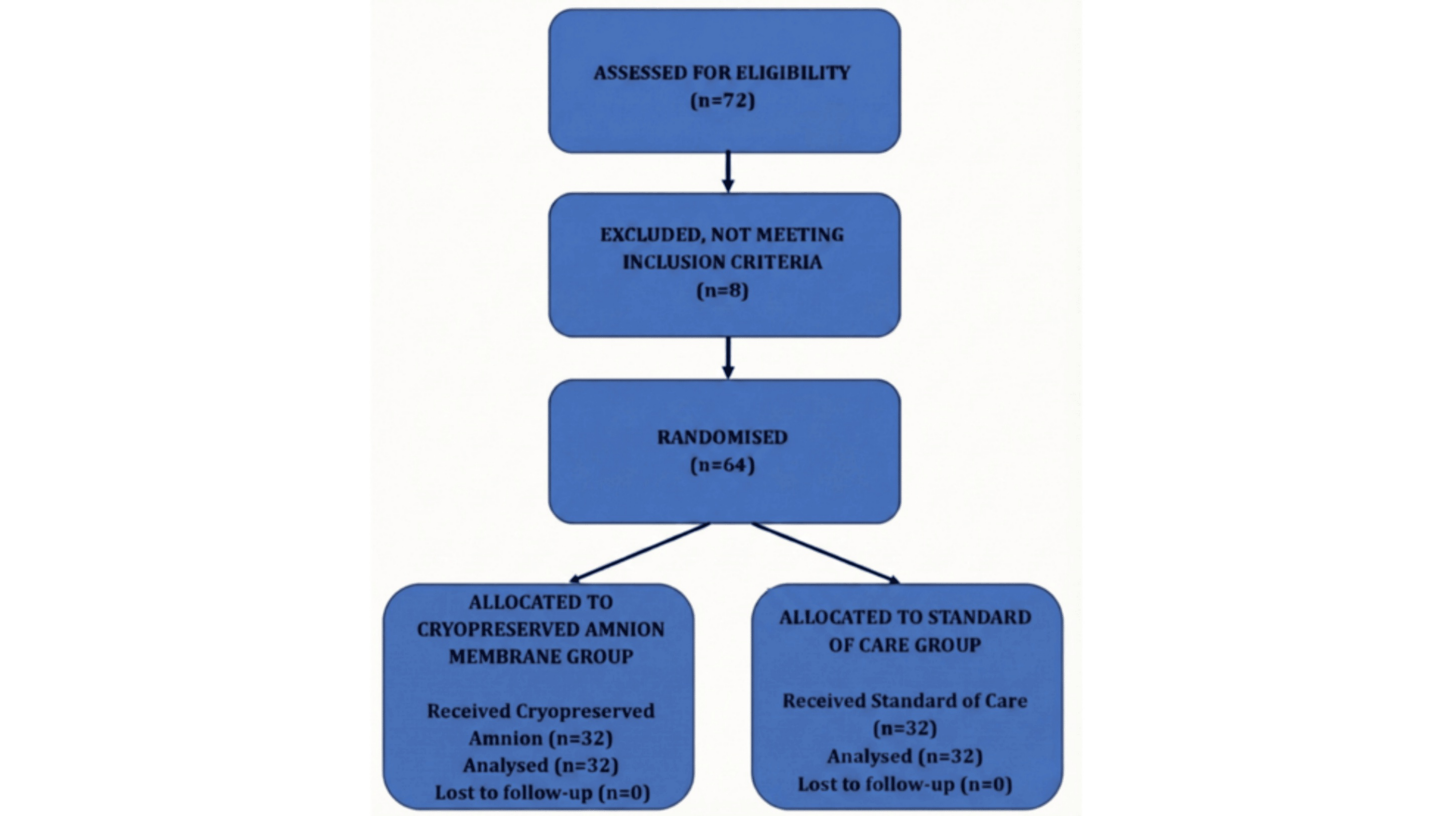
CONSORT flow diagram Flow diagram illustrating the study design, participant enrollment, and allocation. A total of 72 patients were assessed for eligibility, of whom 64 were randomized into the Intervention (Cryopreserved Amniotic Membrane) and Control (Standard of Care) groups. All 64 participants completed the 12-week follow-up and were included in the final analysis. Created by the authors.

A total of 72 patients were screened; 64 were enrolled and randomized (Figure 1).

Baseline characteristics, including age, gender distribution, ulcer duration, and initial wound area, were comparable between the two groups (p>0.05), as detailed in Table 1.

**Table 1:** Baseline demographic and clinical characteristics of the study participants SD: Standard Deviation; n: Number of patients. Data are presented as Mean ± SD or frequency (%). Statistical analysis performed using Student's t-test for continuous variables and Chi-square test for categorical variables (significance set at p<0.05). Data derived from the current study.

Characteristic	Group A (Amnion) (n=32)	Group B (Standard of Care) (n=32)	p-value
Age (years), Mean ± SD	54.2 ± 8.4	56.1 ± 7.9	0.34
Gender, male n (%)	28 (87.5%)	27 (84.4%)	0.71
Ulcer duration (months)	14.2 ± 4.5	13.8 ± 5.1	0.68
Initial wound area (cm²)	12.4 ± 3.6	11.9 ± 4.1	0.59

Wound healing efficacy

**Figure 2:**
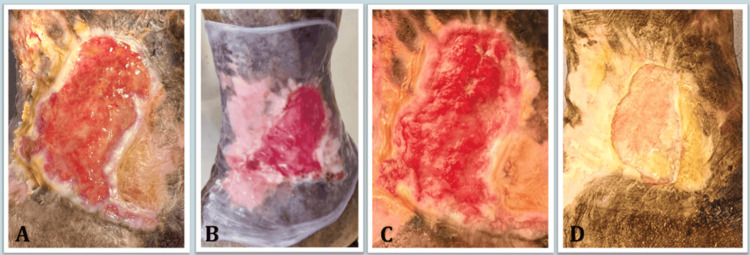
Clinical healing progression Photographic series demonstrating the healing progression of a chronic venous ulcer treated with cryopreserved amniotic membrane. (A) Baseline presentation at Day 0 showing a large, non-healing ulcer with slough. (B) Intra-operative view showing the application of the amniotic membrane graft over the wound bed. (C) Assessment at Day 5, revealing graft integration and granulation. (D) Outcome at Day 24 showing wound area reduction and epithelialization from the margins. Images are from the current study.

The intervention group demonstrated progressive clinical healing trajectories over the observation period (Figure 2).

At the 45-day primary endpoint, complete wound closure was observed in 26 (81.25%) patients in the cryopreserved amniotic membrane group, compared to 15 (46.88%) patients in the control group. The absolute risk difference for wound closure was 34.4% (95% CI: 12.4% to 56.3%; Fisher’s exact test, p=0.004).

**Figure 3:**
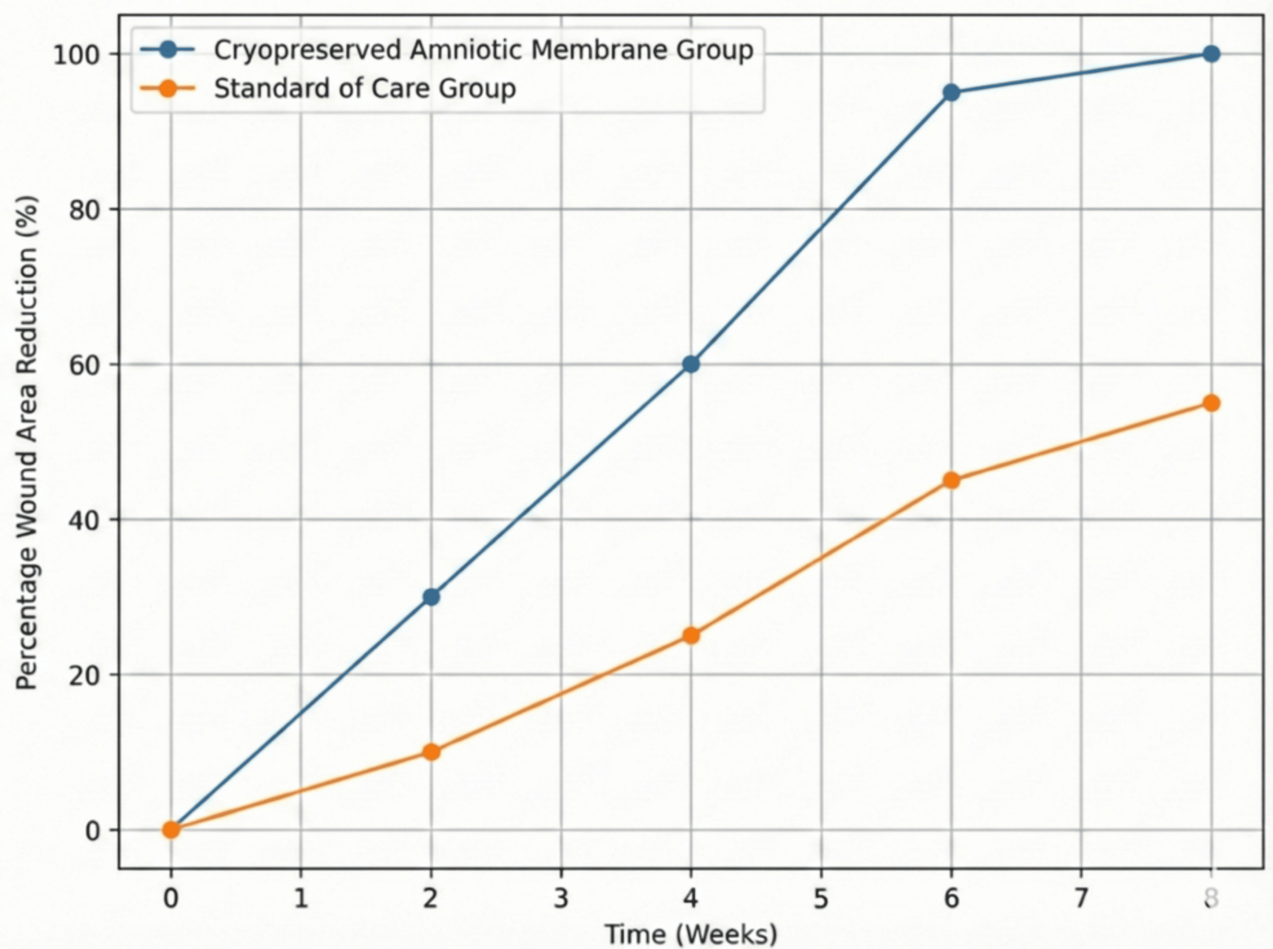
Comparative wound area reduction Line graph comparing the mean percentage reduction in wound area over time between the Cryopreserved Amniotic Membrane group and the Standard of Care group. The blue line represents the Cryopreserved Amniotic Membrane group, and the orange line represents the Standard of Care group. Data derived from the current study.

The mean percentage reduction in wound area by day 21 was 64.5% for the intervention group versus 28.2% for the control group (Figure 3).

**Figure 4:**
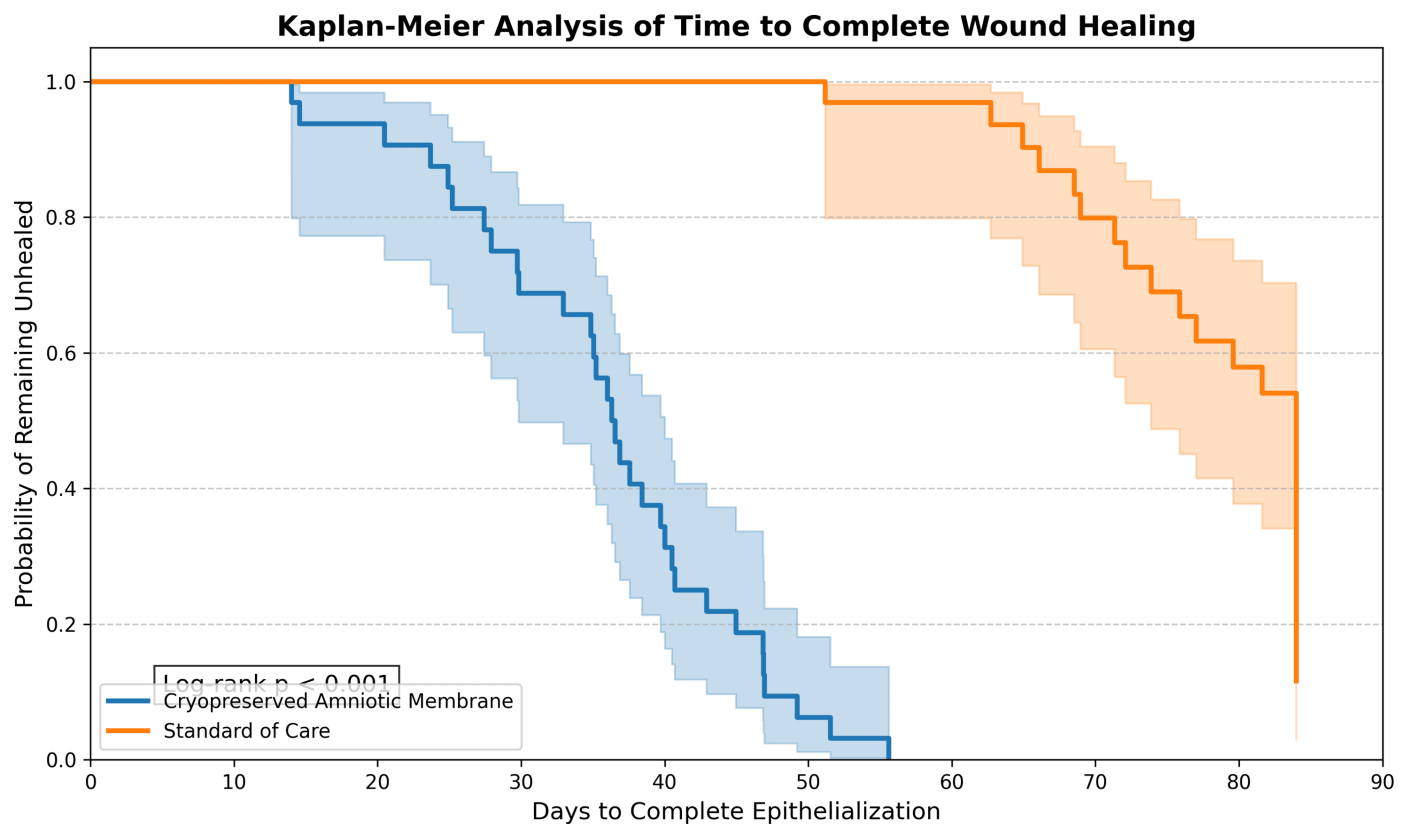
Kaplan-Meier survival analysis for time to complete wound healing Kaplan-Meier survival curves comparing the probability of complete wound closure over the 12-week follow-up period. The blue line represents the Cryopreserved Amniotic Membrane group (median time to healing: 36 days); the orange line represents the Standard of Care group (median time to healing: 78 days). Statistical significance (Log-rank test, p < 0.001) is indicated. Data derived from the current study.

Time-to-event analysis using Kaplan-Meier survival estimates(Figure 4) indicated a lower median time to complete epithelialization in the intervention group. The median time to complete healing was 36 days (95% CI: 31 to 41 days) for the intervention group compared to 78 days (95% CI: 71 to 85 days) for the control group (Log-rank test, p < 0.001).

Pain relief and complications

**Figure 5:**
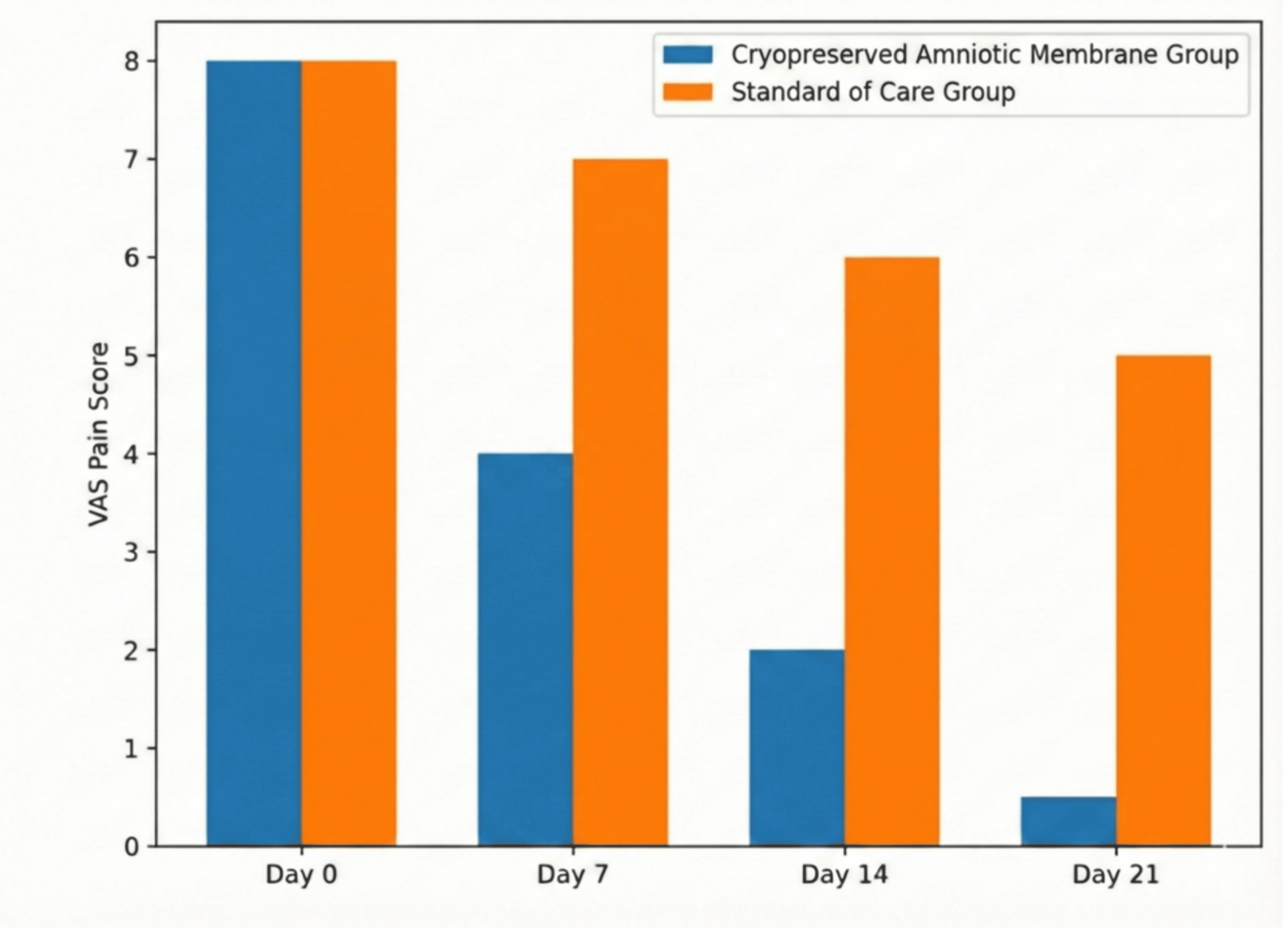
VAS pain score analysis Clustered bar chart illustrating the mean pain intensity scores as measured by the Visual Analog Scale (VAS) at baseline and follow-up intervals for both the study groups. Blue bars represent the Cryopreserved Amniotic Membrane Group, and orange bars represent the Standard of Care Group. Data derived from the current study.

Pain scores decreased in the intervention group. Mean VAS scoresdeclined from a baseline of 7.8 ± 1.2 to 2.1 ± 0.9 by day 14 (Figure 5). The control group reported a change in pain scores from 7.6 ± 1.4 to 5.4 ± 1.1 over the same period (Mann-Whitney U test, p<0.05).

The incidence of microbiologically confirmed wound infection was 6.25% (two patients) in the intervention group compared to 28.12% (ninepatients) in the control group. The risk difference for infection was 21.9% (95% CI: 4.4% to 39.4%; Fisher’s exact test, p=0.02). No cases of graft rejection or systemic adverse events were recorded during the 12-week follow-up.

## Discussion

The clinical application of human amniotic membrane dates to the early 20th century [13,14]. Literature records its initial use in addressing pain and infection, attributes linked to the tissue's molecular composition. Its utility has subsequently expanded across various surgical disciplines [15].

The current study indicates that the application of cryopreserved amniotic membrane reduces healing time in recalcitrant venous ulcers compared to standard compression therapy. These findings are consistent with literature investigating the regenerative properties of amniotic tissue [16-18]. The theoretical advantage of cryopreserved variants relies on the preservation of the native extracellular matrix (ECM) and biological signaling molecules, avoiding proteomic alterations associated with heat dehydration. Cryopreserved amniotic membrane maintains the histological structure and the heavy chain-hyaluronic acid/pentraxin 3 (HC-HA/PTX3) complex, which is hypothesized to suppress inflammatory cell migration and inhibit fibrosis [10].

The granulation tissue observed may be facilitated by growth factors within the graft acting as a scaffold for keratinocyte migration [19,20]. Additionally, the reduction in median healing time is consistent with the hypothesis that the stromal matrix may sequester circulating progenitor cells, thereby modulating the chronic wound microenvironment [21].

A decrease in VAS pain scores was recorded in the intervention group. This observation aligns with proposed neuro-modulatory mechanisms of the amniotic membrane, which covers exposed nerve endings and is theorized to downregulate inflammatory cytokines [5]. Pain alleviation correlated with patient compliance regarding compression therapy.

Cost analysis and economic implications

Regarding economic parameters in the Indian healthcare setting, the preparation and supply of a standard 5x5 cm cryopreserved amniotic graft cost approximately ₹2,000 (Indian Rupees) in this study. The intervention group achieved a median healing time of 36 days compared to 78 days for the standard of care. This 42-day differential theoretically offsets indirect clinical costs, including the continuous requirement for dressing materials, nursing care, and outpatient hospital visits [22]. Therefore, the initial biological dressing cost may be balanced by a reduction in long-term resource utilization.

Limitations

The limitations of this study include its single-center design, reliance on convenience sampling rather than an a priori power calculation, and the inability to blind the operating surgeons to the intervention. The study cohort presented an 87% male proportion, which represents a selection bias reflecting the occupational demographics of the referral center; this limits the generalizability of the findings to female populations. Future multi-centric studies with larger, gender-balanced cohorts are required [23]. Furthermore, the protocol utilized cryopreservation at -80°C. While this maintains cellular viability [24], it necessitates cold-chain logistical coordination, potentially restricting adoption in resource-limited peripheral clinics compared to shelf-stable dehydrated alternatives.

## Conclusions

Cryopreserved amniotic membrane demonstrates efficacy in promoting wound closure, lowering infection rates, and reducing pain in patients with chronic venous leg ulcers. Supported by time-to-event survival analysis, its application serves as an evidence-based biological adjunct. Despite logistical requirements regarding cold-chain storage and demographic limitations within the cohort, cryopreserved allografts provide a measurable clinical benefit in the management of venous ulcers resistant to standard compression therapy.
